# Scribbling the Cat: A Case of the “Miracle” Plant, *Moringa oleifera*

**DOI:** 10.3390/plants8110510

**Published:** 2019-11-15

**Authors:** Thulani Tshabalala, Bhekumthetho Ncube, Ntakadzeni Edwin Madala, Trevor Tapiwa Nyakudya, Hloniphani Peter Moyo, Mbulisi Sibanda, Ashwell Rungano Ndhlala

**Affiliations:** 1 Agricultural Research Council (ARC), Vegetable and Ornamental Plants (VOP), Private Bag X923, Pretoria 0001, South Africa; tshabalalat1@arc.agric.za (T.T.); ncubeb@arc.agric.za (B.N.); 2 School of Agricultural, Earth and Environmental Sciences, University of KwaZulu-Natal Pietermaritzburg, Private Bag X01, Scottsville 3209, South Africa; sibandam3@ukzn.ac.za; 3 Department of Biochemistry, School of Mathematical and Natural Sciences, University of Venda, Private Bag X5050, Thohoyandou, 0950, South Africa; ntaka.madala@univen.ac.za; 4 Department of Physiology, School of Medicine, Faculty of Health Sciences, University of Pretoria, Pretoria, South Africa; trevortn@gmail.com; 5 Department of Human Anatomy and Physiology, Faculty of Health Sciences, University of Johannesburg, Doornfontein, Johannesburg 2002, South Africa; 6 Agency for Technical Cooperation and Development (ACTED), Amman, Jordan; hmthunzi@gmail.com; 7 Department of Life and Consumer Sciences, College of Agriculture and Environmental Sciences, University of South Africa, Private Bag X6, Florida 1710, South Africa

**Keywords:** allelopathy, glucomoriginin, glucosinolates, invasive species, Moringaceae

## Abstract

This paper reviews the properties of the most cultivated species of the Moringaceae family, *Moringa oleifera* Lam. The paper takes a critical look at the positive and the associated negative properties of the plant, with particular emphasis on its chemistry, selected medicinal and nutritional properties, as well as some ecological implications of the plant. The review highlights the importance of glucosinolates (GS) compounds which are relatively unique to the *Moringa* species family, with glucomoriginin and its acylated derivative being the most abundant. We highlight some new research findings revealing that not all *M. oleifera* cultivars contain an important flavonoid, rutin. The review also focuses on phenolic acids, tannin, minerals and vitamins, which are in high amounts when compared to most vegetables and fruits. Although there are numerous benefits of using *M. oleifera* for medicinal purposes, there are reports of contraindications. Nonetheless, we note that there are no major harmful effects of *M. oleifera* that have been reported by the scientific community. *M. oleifera* is suspected to be potentially invasive and moderately invasive in some regions of the world because of its ability to grow in a wide range of environmental conditions. However, the plant is currently classified as a low potential invasive species and thus there is a need to constantly monitor the species. Despite the numerous benefits associated with the plant, there is still a paucity of data on clinical trials proving both the positive and negative effects of the plant. We recommend further clinical trials to ascertain the properties associated with the plant, especially regarding long term use.

## 1. Introduction

Up to about 80% of the world’s population use natural remedies such as herbs for medication, mainly because of the ease of accessibility, affordability and most of all, because of safe therapeutics [1]. Traditionally, plants produce secondary metabolites as an adaptive defence mechanism against a broad spectrum of potentially damaging biotic and abiotic factors such as pathogens and the environment. Humans have thus exploited the properties of this biogenic resource (secondary metabolites) in their fight against human pathogenic microbes [2]. Through their diverse chemical structures, man has explored and exploited plant secondary metabolites beyond their obvious antimicrobial properties, especially for human conditions such as cancer, diabetes, inflammation, cardiovascular, etc. The past decades have seen several plants exploited for their phytoconstituents in either the development of medicine or nutritional purposes. One such plant has been *Moringa oleifera* Lam., which is known to possess a wide spectrum of metabolites with purported nutritional and medicinal properties [3]. This plant is commonly known as the “miracle tree” due to its purported healing powers across the different spectrum of diseases.

*Moringa oleifera* belongs to the Moringaceae family (order Brassicales). The Moringaceae family has a total of 13 species and *M. oleifera* is the most utilised and cultivated species [4,5]. The plant is naturally occurring in the north-eastern parts of India. However, due to its ability to grow in a wide range of conditions, it is now widely cultivated in the tropic and subtropical regions of the world [4]. The plant can grow up to 2 m in the first year and up to 12 m when mature and bears long, drumstick shaped pods within the first year [6,7]. The common names given to this tree include horseradish tree because of the taste of the roots and drumstick tree because of the shape of the fruits pods on the tree. The plant is referred to as the “miracle tree” because of the enormous positive impact it has on people’s livelihoods. It is reported that every plant part (seeds, flowers, stems, leaves and roots) is a great source of nutrients and produces major essential medicinal principles [8,9,10,11], curing a range of diseases [12,13]. For industrial uses, biodiesel and cosmetic oil can be extracted from the seeds. The seeds can also be used in water purification processes [14,15].

*Moringa oleifera* is one of the plants with a great phytochemical profile, and it is considered to be in the top 10% out of 500,000 species being used for conventional medicines [16]. With so much attention given to the plant, there is a need to review the literature on what has been documented thus far. In particular, we review the positive and associated negative properties of the plant. In doing so, we specifically looked at the chemistry, medicinal and nutritional properties, as well as the ecological implications of the plant. This review sought to collate the important research findings reported on *M. oleifera* to date, enable researchers to identify the existing research gaps and allow the industry to explore the collated information in developing new products.

## 2. Phytochemicals in *Moringa oleifera*

Plants are devoid of mobility to defend themselves from external stressful conditions, and they have instead evolved and armed themselves with several secondary metabolites to counter stress from temperature, water, light intensity, herbivory and microbial attack [17]. *M. oleifera* was initially introduced to various communities with very little knowledge about its chemistry. However, in recent years, various studies have reported the plant as a reliable source of potential health-improving chemicals. As expected, the chemistry of *M. oleifera* is interesting, comprising of different classes of compounds. Moreover, the plant has been shown to have advanced biosynthetic pathways which ultimately result in a diversified chemical profile [18]. To date, various compounds such as glucosinolates [19], flavonoids [18], phenolic acids [20], and other compounds found in *M. oleifera* have been investigated. 

### 2.1. Glucosinolates

Glucosinolates (GS) are a heterogeneous group of sulfur and nitrogen containing glycosidic secondary metabolites [21,22]. GS are secondary compounds relatively unique to the Moringaceae family [19,22] and to the family of Brassicaceae which include cabbage, broccoli and cauliflower. As a group, these compounds are widely spread across different parts of the plant, with the seeds containing the highest concentrations compared to the leaves [23]. These compounds are derived from amino acid precursors and, as such, they can either comprise short- and long-chain aliphatic glucosinolates (Ile, Leu, Val, Ala and Met), indolic glucosinolates (Trp) and aromatic glucosinolates (Tyr and Phe) [4,24,25]. Interestingly, it has been shown that a certain type of the compounds is restricted in a certain parts of the plant, for instance, benzyl glucosinolate (glucotropaeolin) is predominant in the roots [19], whilst glucomoriginin is commonly found in stems, flowers, pods, leaves and seeds [26]. Apart from the intra-plant variation, the levels of glucosinolates have been noted to vary between plants collected from different geographical areas [19]. The most abundant GS molecule in *M. oleifera* is glucomoriginin and its acylated derivative [20]. The acylated isomers of glucomoriginin represent an interesting phenomenon since this could be regarded as an evolutionary strategy by the plant in order to maximize its metabolite composition. This is a phenomenon which was seen with other metabolites such as phenolic compounds in *M. oleifera* [20]. It was also observed that the three acylated isomers of glucomoriginin elute at different chromatographic regions during reverse phase LC-MS analyses, an indication that they differ in terms of polarity [27], which might have effects on the bioavailability. 

In plants, it is believed that GS are biologically active, however, their metabolised products (i.e., isothiocyanates, nitriles, thiocyanates, epithionitriles and oxazolidines) are deemed to be highly active [28,29]. Elsewhere, oxidative stress has been shown to be a potent inducer of GS in plants [27] and, by extension, GS molecules are believed to mitigate effects associated with oxidative stress. As such, the use of GS molecules as direct and indirect antioxidants have been investigated [30]. Interestingly, GS molecules have been shown to possess anti-cancer activities [29]. Protective effects of GS molecules against neurodegenerative diseases has been shown elsewhere [31].

### 2.2. Flavonoids

**Figure 1:**
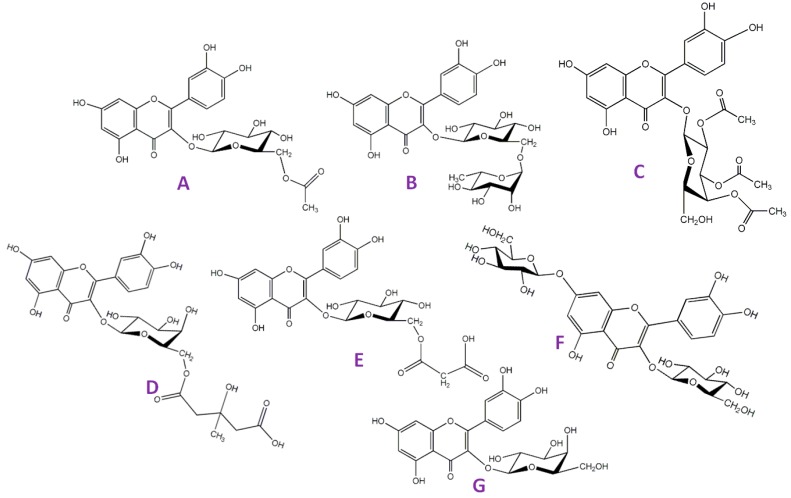
Different structures of *Moringa oleifera* chemical compounds, mainly quercetin glycosides showing different glycosylation pattern. (**A**) quercetin-acetylhexose; (**B**) quercetin-rutinoside; (**C**) quercetin-triacetylhexose; (**D**) quercetin-hydroxy-methylglutaroyl hexose; (**E**) quercetin-malonylhexose; (**F**) quercetin-dihexose; and (**G**) quercetin-hexose.

Similar to the GS molecules, flavonoids are highly active metabolites produced by plants [32]. Consumption of flavonoids has been linked with reduced risks associated with various diseases [33,34]. As such, humans prefer foods rich in these nutraceuticals since their consumption has been positively correlated with the delayed onset of some age-related diseases [35]. Intake of flavonoids has also been shown to have positive effects on cancer-related diseases [36] and as potent anti-inflammatory agents [37]. Elsewhere, these molecules have been shown to have application in the cosmetic and skincare industry [38,39]. *M. oleifera*, like any other plants, produces flavonoids and to date, the chemically diverse profile of flavonoids has been reported in *M. oleifera* [18,40]. Only four types of flavonoids, namely quercetin, kaempferol, isorhamnetin and apigenin, have been reported in the plant, and myricetin has also been reported but with very little convincing analytical data [41]. The chemistry of *M. oleifera* plant is interesting considering the chemical diversity of its flavonoids. Unlike other plants, *M. oleifera* diversifies its flavonoids through complex glycosylation patterns. For instance, when compared to its closely related species, *M. ovalifolia*, it was found that *M. oleifera* produces similar aglycone flavonoids (quercetin, kaempferol and isorhamnetin), but the differences arise when the flavonoid glycosides are concerned. Here, *M. oleifera* is shown to attach different types of sugars on its flavonoid aglycones. Using quercetin as an example (Figure 1) it can be seen that this flavonoid is glycosylated using various types of sugar attachment [18,40]. Interestingly, the same type of glycosylation has been noted in another closely related species, *M. stenopetala* [19]. Some of the sugar modification includes, amongst others, acetyl hexose, malonyl hexose, di-glycosylation, and tri-acylation [40]. As seen with the GS molecules, the attachment of groups such as acetyl on the sugar moiety of the active metabolites changes its polarity and ultimately its bio-availability. Therefore, this type of diversification (through glycosylation) by *M. oleifera* is an indication that the plant can be a useful source of bio-available flavonoid compounds which can be used in physiological environments at different polarities. It is also important to mention that the same glycosylation patterns have been noted with other similar flavonoids such as kaempferol and isorhamnetin [40]. This phenomenon of diversification can be said to be species/genus specific because other plants such as *Vernonia fastigiata* diversify their flavonoid composition by swapping sugar positions [42]. For instance, *V. fastigiata* produces two isobaric molecules, quercetin-3-O-hexoside-O-pentoside and quercetin-3-O-pentoside-O-hexoside, both appearing at m/z 595.1245 but at different retention times, again indicating differences in polarities [42]. As such, the above is an indication that advanced analytical techniques need to be developed in order to fully cover the flavonoid composition of the plant. In another example, a flavonoid peak appearing at m/z 593 with fragment ions at m/z 353 and 473 was once identified as Multiflorin B [40], which was later disputed and re-identified as Vicenin-2 [18], a molecule with potential health benefits [43].

Strangely, several studies have indicated that *M. oleifera* does not produce a highly bio-available flavonoid called rutin. Research conducted on the two species of *Moringa* (*M*. *stenopelata* and *M. oleifera)*, discovered that, though the two plants are genetically related, *M. stenopelata* leaves contained rutin as one of the predominately active compounds in amounts as much as 2.3% of dry leaf weight, which was not found to be present in *M. oleifera* [44]. As indicated above, in a similar study by Makita et al. [18], it was reported that *M. oleifera* contained more flavonoids than *M. ovalifolia.* However, on a close look, all flavonoid compounds in *M. ovalifolia* were shown to be glycosylated only with rutinoside sugar. From the two studies above, it was concluded that *M. oleifera* is incapable of glycosylating its flavonoids with rutinoside sugar. However, in a twist of fate situation, in a follow-up study by Makita et al. [45], comparing 12 cultivars of *M. oleifera* indicated that some of the cultivars contained rutin and it was concluded that the presence of rutin is a cultivar-specific phenomenon, with 3 out of 12 cultivars of *M. oleifera* able to carry out this glycosylation. Therefore, rutinoside-bearing flavonoid occurrence is cultivar-specific and as such, offers differences in pharmacological potency of plants. This phenomenon might, however, result in negative perception towards *M. oleifera* as a nutraceuticals source, as one has to be sure if the cultivar they are planting/processing contains rutin.

### 2.3. Phenolic Acids

Similar, to flavonoids, *M. oleifera* contains a large contingency of phenolic acid derivatives with purported biological activities. From the leaf extracts, various isomers of chlorogenic acids (CGAs) have been identified [40,45]. CGAs are ubiquitous and indispensable phenolic compounds found in various plants [46], and are formed as a result of esterification between various forms of cinnamic acids and a quinic acid molecule [45]. Due to the structural orientation (stereochemistry) of the quinic acid, different isomers of CGAs can be formed and these differ from one plant to another [47] and to date, coffee beans have been found to contain the largest composition of the compounds [48]. However, LC-MS based analyses of 80% methanolic extracts of *M. oleifera* leaves revealed that the plant contains structurally diverse chlorogenic acids such as caffeoylquinic acids, feruroylquinic acids and coumaroylquinic acids [45]. Each of these class of compounds was found as a group of isomers, for instance, more than four peaks of caffeoylquinic acids appearing at m/z 353, represented as positional isomers and geometrical isomers thereof. The former is believed to be formed enzymatically [46] whilst the latter are believed to form as a result of UV exposure (in a form of sunlight), even though, elsewhere, they were shown to form as a result of metabolic activity associated with a defensive mechanism of a plant [49]. Chlorogenic acids, in general, are known to offer protection against oxidative stress-related diseases. To substantiate the above, the levels of different positional and geometrical isomers of CGAs were found to be perturbed by oxidative stress induced by gamma radiation of *M. oleifera* leaves [20]. Such findings gave an indication that geometrical isomers of this plant are not just mere structural artefact, but biologically active compounds. According to Ramabulana et al. [20], the structurally diverse composition of CGA molecules found in *M. oleifera* could be an evolutionary strategy to maximize biologically active molecules through isomerization with an intention to increase its defensive chemical arsenal against various stressors. This phenomenon demonstrates the “better be ready than sorry” phenomenon which states that plants create a defensive environment by producing a large contingency of structurally diverse defence compounds (phytoanticipins and phytoalexins) in order to strengthen their innate immunity to be deployed against various forms of stress. Therefore, the constitutive presence of CGA molecules in *M. oleifera* plants offers an overwhelming pharmacological advantage, since these compounds have been associated with beneficial health attributes [48].

The amount of phenolic acid in *M. oleifera* varies depending on several factors, such as the rainfall received. Water deficiency in plants results in oxidative stress and the plants, in turn, respond by increasing production of antioxidant compounds [50]. This was confirmed by Leone et al. [51], who observed that *M. oleifera* plants grown in water stressful environments had greater amounts of total phenolics and antioxidant capacities. The plant part also plays a role, as leaves have higher amounts of phenolics when compared to roots [52]. Other factors affecting phenolic acid concentration include the harvesting stage of the plant, cultivar of *M. oleifera* and the extraction method used [53,54,55]. Free hydroxyl compounds contained by the phenolics found in *M oleifera* leaves are responsible for reduction reactions which aid in the prevention of degenerative diseases such as diabetes [56,57].

### 2.4. Vitamins and Minerals

*Moringa oleifera* has been described as the most nutritious tree yet discovered [58]. We summarise some of the nutritional properties of the plant in Table 1. 

### 2.5. Tannins

Tannins are water-soluble polymeric phenolics that bind to proteins and alkaloids [66]. The amount of tannins in *M. oleifera* range between 13.2 g (tannin acid equivalent) TAE/kg and 20.6 g/kg in air-dried leaves [67,68]. In leaves, they contribute to about 3.2% of dry matter [69]. However, the roots of the *M. oleifera* have more of condensed tannins (proanthocyanidins) than leaves, which results in the roots having a higher antioxidant activity [52]. This is because tannins are involved in the reduction of peroxyl radicals due to hydroxyl groups (OH-) [70]. Furthermore, the tannins in *M. oleifera* have been reported to contribute to anti-cancer, antimicrobial and anti-hepatoxic activity [55]. However, in animals such as goats, tannins from trees such as *Vachellia nilotica* have been reported to reduce feed intake, nutrient digestibility and nitrogen retention [71], because of the astringent taste and their ability to precipitate proteins which renders them indigestible. Lu et al. [72] reported that inclusion of *M. oleifera* leaf meal (≥10%) in poultry diets resulted in reduction in egg weight due to tannins causing lower protein retention and digestibility. Therefore, some researchers have recommended less than 10% inclusion of *M. oleifera* in poultry diets, with no effects on the feed intake [73].

## 3. Nutritional Aspects of *Moringa oleifera*

### 3.1. In Humans 

In addition to its abundant supply of bioactive phytochemicals that are important in ethnomedicinal management of diseases, *M. oleifera* is also commonly utilised as a food crop, thus making it a functional crop [74]. Due to its high drought and disease resistant properties, *M. oleifera* is often used as a famine food in several African communities [75,76]. The use of *M. oleifera* as a food crop by humans is supported by the fact that its leaves are an abundant source of polyunsaturated fatty acids (PUFAs) such as omega-3 (ω-3) and omega-6 (ω-6) apart from the micro-elements and protein qualities [77], which are important in vitalising the body and in cardiovascular function. *M. oleifera* pods and flowers have a high content of total monounsaturated fatty acids while the seeds and oil from the seed possess a high content of oleic and palmitoleic acid [78]. Oleic and palmitoleic acid are important in lowering plasma cholesterol levels and ameliorating the effects of diabetes and insulin resistance [79]. Generally, the different parts of *M. oleifera* possess low saturated fatty acid (SFAs), high monounsaturated fatty acids (MUFA) and PUFA content that can be useful for human health, especially if food is supplemented and fortified with *M. oleifera*. 

As mentioned above, different parts of the *M. oleifera* plant are rich in mineral content (micro-elements) such as potassium (K), iron (Fe), calcium (Ca) and magnesium (Mg) [80]. Human consumption of *M. oleifera* can thus be beneficial in preventing negative health outcomes associated with mineral deficiencies. Echoing the above, *M. oleifera* kernels are rich in proteins [68] and as such, can be used as a good source of protein particularly for human food product formulation and supplementation. Experimental proximate studies have also shown that the *M. oleifera* leaf powder consists of carbohydrates and proteins [68] that can be used to increase the nutritional value of staple foods fortified with the *M. oleifera* foliage. Inclusion of more than 1% weight for weight (w/w) of *M. oleifera* leaf powder has shown to result in reduced acceptability due to bitterness associated with the plant [81]. Despite its widespread potential as a nutritional supplement for human consumption, there is a need to perform further in vitro and in vivo experimental studies on the bioavailability and digestibility of nutrients in *M. oleifera.*

### 3.2. In Livestock

*Moringa oleifera* is a multiple purpose tree that is used as a medicinal plant, spice, and food, among other uses. In addition to its use in ethnomedicine, *M. oleifera* has several agricultural applications. It has successfully been used as a fertiliser and a natural biopesticide against several plant pathogens [82]. Nutritional analyses of *M. oleifera* leaves and seeds have shown that they possess a high protein content, carotenoids, minerals and vitamins [69]. The presence of high protein content and other important nutrients [69,83] makes *M. oleifera* leaves an important contributor to livestock feed quality and quantity [83]. Vitamins, in particular, contribute significantly to the immune systems of the animals thus preventing the development of several diseases in the livestock [8]. The high nutritional value of *M. oleifera* seeds and leaves makes it suitable for livestock feed supplementation to either improve the growth performance of the livestock or replace traditional crops and provide an economically sustainable source of feed [82]. Previous studies have shown that supplementing livestock with feed containing *M. oleifera* leaves improves digestibility [84], and confers beneficial effects on growth and carcass characteristics of animals whose diet is supplemented with *M. oleifera* [82].

Due to droughts and low rainfalls recorded in most tropical regions, supplying feed for livestock by farmers has increasingly become a major challenge, particularly during dry seasons. In some southern African regions that are characterised by harsh climatic conditions such as the Limpopo Province in South Africa, smallholder farmers are being encouraged to cultivate *M. oleifera* to supplement their livestock feed due its high nutritional value and its cash-earning potential [76]. The *M. oleifera* seed also has a high oil content [85] and may be used for both livestock and human consumption.

The use of *M. oleifera* as a nutritional supplement in animal feed must be done with caution. Some parts of the *M. oleifera* plants, such as the leaves and bark, have antinutritional factors and there is a limit, not being in excess of more than 10% (w/w) of the diet [73].

## 4. Medicinal Properties of *Moringa oleifera*

As a medicinal plant duped the ‘miracle tree’, *M. oleifera* is used extensively and broadly in a number of ailments, most of which have been tested pharmacologically and clinically in various mechanistic and animal models. It is thus outside the scope of this review to exhaustively discuss this extensive list of studies done on *M. oleifera* extracts, but we thus limit our discussion to highlighting a selected few medicinal properties of *M. oleifera* extracts.

### 4.1. Antioxidant Properties

Extracts from the different solvents and plant parts of the *M. oleifera* are known to possess antioxidant properties [86]. Leaf methanol and ethanol extracts, in particular, have shown some scavenging properties towards superoxyl and peroxyl radicals [86,87]. Using a UV accelerated method at 50 °C, *M. oleifera* seed oil fraction was evaluated for their protection against rancidity to fresh sunflower oil and demonstrated superior antioxidant properties. When these activities were compared with those of α-tocopherol and BHT (common synthetic antioxidant agents) using the same method on the same sunflower oil, *M. oleifera* seed oil fraction exhibited higher antioxidant activity than the two known agents [88]. In another study, aqueous ethanolic extracts of *M. oleifera* leaf and flower, using biochemical oxidative tissue markers, led to a significant decline in rat liver damage compared to the control treatment [89]. The seed, fruit and leaf aqueous *M. oleifera* extracts were examined for their potential to prevent DNA oxidative damage as well as their antioxidant properties. The results revealed that these extracts have a significant potential of inhibiting DNA damage as well as synergistically inhibit with trolox, in an effective sequence of leaf > fruit > seed [90].

An experimental study on goats fed with leaf extracts of *M. oleifera* showed that treatment with *M. oleifera* reduced lipid peroxidation and increased the antioxidant activity of glutathione peroxidase and catalase [91]. Thiobarbituric acid reactive substance (TBARS) values were significantly lower in chicken sausage samples incorporated with 0.5%, 0.75% and 1% *M. oleifera* leaf powder, compared to the negative and the positive (BHT) controls throughout the five weeks storage duration at 4 °C [92]. On the other hand, Hazra et al. [93] reported a comparably lower TBA value of buffalo meat supplemented with 1.5% *M. oleifera* leaf extract than those of the control treatment. Compared to the control, the leaf extracts (0.1%) were shown to magnificently reduce lipid oxidation in cooked patties of goat meat [94]. Similar trends are also reported in recent research findings [95], where goat meat fed with meal supplemented with *Moringa oleifera* leaf or sunflower cake (SC) or grass hay (GH) were compared for their antioxidant properties. Moringa-supplemented meat had higher scavenging potential towards ABTS (93.5%) and DPPH (59%) than the other two meal supplements. These results can be attributed to the inhibition of lipid peroxidation by the antioxidant phytochemical agents found in *M. oleifera* leaves such as polyphenols. Literature is replete with numerous other findings on the antioxidant properties of *M. oleifera* extracts and thus cannot be overemphasised here. It is prudent to mention that, of the most literature reviewed, phenolic acids and flavonoids feature prominently as responsible agents for most of the reported antioxidant activities (Table 2).

### 4.2. Anti-Inflammatory Properties

Inflammation is one of the key characteristics of the diseases that result from the tilted balance of anti-inflammatory cytokine regulated by T helper cells [96]. Type 2 diabetes, a result of metabolic dysfunction, is linked to elevated levels of systemic pro-inflammation markers [97]. Diabetic patients exhibit elevated levels of both TNF-α and IL6 contributing to the advancement of micro and macrovascular changes, which is a characteristic of diabetic patients. The seeds and pods of *M. oleifera* have been highlighted in numerous studies as having positive anti-inflammatory properties [98,99,100]. The root extracts of *M. oleifera* were reported to have acute anti-inflammatory properties in a carrageenin-induced rat paw oedema test [101,102]. An evaluation of the stem bark extracts for their immunomodulation properties on human monocyte cells (THP-1) revealed substantial inhibition of pro-inflammation cytokines (TNF-α, IL-6, and IL-1β), as well as reactive oxygen species (ROS) and nitric oxide (NO) production [103]. Eicosane, cis-13-octadecenoic acid, hexadecane, benzoic acid, n-hexadecanoic acid, heptadecane, dodecane, hexadecanoic acid, methyl ester, β-sitosterol and ethyl ester are some of the metabolites identified in root, leaf and seeds of *M. oleifera* [4,104,105], and some of which are known for their anti-inflammatory properties [106,107,108]. The anti-inflammatory properties of *M. oleifera* confirm the ethnomedicinal uses of the plant to treat various diseases that are associated with inflammatory processes. 

### 4.3. Anti-Diabetic Properties

Metabolic syndrome (MetS) comprises of a cluster of risk factors that are related to glucose and lipid metabolism (obesity) as well as cardiovascular dysfunction (blood pressure) [109,110]. Recently, the prevalence of MetS health outcomes such as Type 2 diabetes are on the rise, posing a public health burden particularly in the developing world [111]. This has necessitated the need to explore the therapeutic efficacy of alternative and complementary treatments.

Experimental animal models reveal that orally administered *M. oleifera* leaf extract reduces the progression of fructose-induced diabetes [112]. Later studies show that *M. oleifera* leaf powder ameliorates alloxan-induced hyperglycemia [113], indicating its potential in managing diabetes. These anti-diabetic properties of *M. oleifera* were further demonstrated in an animal study that revealed aqueous leaf extracts normalising diet- and streptozotocin-induced hyperglycaemia and hyperinsulinaemia [114,115]. Jaiswal et al. [116] assessed the variable doses of the aqueous *M. oleifera* leaf extract on their anti-diabetic potential on mildly- and severely-induced diabetic rats. Decreased levels of glucose (29.9%) were recorded from normal rats administered with 200 mg/kg of *M. oleifera*. In cases of severely diabetic rats, glucose levels were brought to near normal levels with a reduction of 69.2% and 51.2%. An accompanying improvement in total protein and haemoglobin levels was also reported after 21 days of treatment with *M. oleifera* and thus favourably reducing diabetes [116,117,118]. The hypoglycemic effect of *M. oleifera* extract in this study was found to be comparably similar to Glipizide (an anti-diabetic drug). This experimental evidence further attests to the purported potential of *M. oleifera* extracts in managing diabetic conditions. N-Benzyl nitriles, benzyl thiocarbamates, a benzyl ester and N-benzyl carbamates from the fruit powder extract of *M. oleifera* were reported to have expressively stimulated insulin generation in the beta cells of rodent pancreas. The released insulin had lipid peroxidation and cyclooxygenase enzyme inhibitory properties [119].

Hyperglycaemia emanating from either insulin action orbits abnormal production results in renal, cardiovascular, to ocular complications [117]. This prompted the use of natural medicines in the management of diabetes [118]. For example, the use of *M. oleifera* aqueous leaf extract over a 2-month period re-established all the changes (body weight, plasma glucose, insulin and lipid profile) to normal/near normal on Type 1 diabetic rats [114]. A study by Divi et al. [114] reports *M. oleifera* aqueous leaf extracts to have potent antihyperlipedemic and antihyperglycemic properties on Type 1 and Type 2 diabetic rats. When the anti-diabetic effects of aqueous extract of *M. oleifera* leaves were analysed in histomorphometrical, ultrastructural and biochemical studies by Yassa et al. [120], the altered FPG levels were reduced more than 2-fold and lowered malondialdehyde (>3-fold) and glutathione (>3-fold). The damage to the islet cells was also reported to be reversed significantly. The extract also led to a significant increase (31%) in the area with purple-modified stained β-cells while decreasing (79%) the percentage area of collagen fibres in comparison to the control. Comparable results were also reported by [121]. The major contributors to the progressive development and complications of diabetes were that the weakened antioxidant defence systems prolonged oxidative stress, as well as lipid peroxidation [122].

Improved glucose tolerance through the use of *M. oleifera* supplementation over extended periods has been reported [123]. Gupta et al. [124] provide some plausible meaning and explanation to these results when they discovered bioflavonoids in *M. oleifera,* which plays a crucial role in the uptake of glucose in marginal tissues as well as regulating carbohydrate metabolism. The constituent metabolites in *M. oleifera* enhance the secretion of insulin from β-cells. Bernal-Mizrachi et al. [125] report a marked decline in immune-stained β-cells in diabetic rats. β-cells are the most abundant cells of the endocrine pancreas according to Ross et al. [126], and it is the site where biochemical and histological changes occur during short term treatments with *M. oleifera* leaf extracts [116,127,128,129].

### 4.4. Anti-Cancer Properties

Despite the progress made in the development of chemotherapy in treating cancer, adverse effects such as skin irritation, nausea, nephrotoxicity, infertility, anaemia, and hair loss still exist [130]. It is for this reason that the natural plant-derived anti-cancer sources of treatment with limited side effects are critical in the search for alternative cancer treatments. The potential of *M. oleifera* extracts to treat cancer have previously been demonstrated [131,132,133]. In lung cancer cells, the aqueous fraction of *M. oleifera* leaf extract is reported to have induced an apoptotic effect on HepG2 cells [134]. Leaf extracts administered orally led to a significant reduction (52%) in the proliferation of HepG2 cells and lung cancer cells [135]. One of the advantages of oral cancer therapy is that it leads to the prolonged exposure of the cancer cells and the surrounding environment to the cytotoxic agents. 

A thiocarbamate, niaziminin, derived from *M. oleifera* leaf, is structurally strict for the inhibition of tumour-promoter-induced Epstein-Barr virus (EBV) activation [131]. Studies on the structure-activity relationship showed that an acetoxy group at the 4’-position of niaziminin is an indispensable property for tumour inhibition [136]. A related study by Jung [135], revealed that the aqueous *M. oleifera* leaf extract (300 µg/mL) markedly decreased tumour cell growth, reduced internal ROS level as well as inducing apoptosis in lung and other cancer cell types. Furthermore, *M. oleifera* extracts led to the down-regulation of 90% of the tested genes by margins greater than 2-fold in comparison with the non-treated cells. The authors concluded that the down-regulation of these genes was due to abnormal RNA as a result of *M. oleifera* leaf extract treatment. Vasanth et al. [137] reported *M. oleifera* stem bark extract facilitated silver nanoparticles (AgNPs) as exhibiting exceptional anti-cancer properties on HeLa cells. These AgNPs are reported to exert their activity by increasing ROS production and subsequently inducing apoptotic effect through inhibition of cell replication. 

## 5. Side Effects of *Moringa oleifera*

Although more benefits of using *M. oleifera* for medicinal purposes overshadow its known harmful effects, there are suggestions that it cannot be used in combination with other modern medicines in humans. For example, anecdotal evidence suggests that when treating thyroids, *M. oleifera* compounds in the leaf may aid thyroid function [138]. This evidence further suggests that it can possibly conflict with other thyroid medication triggering drug interaction. It is perceived that *M. oleifera* could adversely slow down the breaking down of substances in the liver [139,140,141]. In that regard, *M. oleifera* could reduce the process of breaking down some medication in the liver. This could progress to cirrhosis and liver failure resulting in malnutrition and weight loss, as well as decreased cognitive function. In addition, *M. oleifera* has been noted to be a good regulator of insulin [142]. Therefore, patients suffering from lack of insulin are bound to have adverse reductions in their sugar levels when using *M. oleifera* for medicinal purposes [141,142]. It is hypothesised that it could decrease the blood sugar to even lower levels when used in combination with other modern medications [141].

A study by Barichella et al. [143] assessed the use, acceptability and safety of *M. oleifera* on children in Zambia. With regards to safety concerns, supplementation of 14 g per day of *M. oleifera* powder was deemed safe for children and adolescents both in the short and long term. Barichella et al. [144] also noted that mild nausea was reported in 20% of the children at various age groups when meals were supplemented with 20 g of *M. oleifera* daily. These side effects were deemed acceptable by the Ethics Committee [143]. Overall, the findings of this study underscore the fact that despite the lack of safety information on the utility of *M. oleifera*, there are no scientifically proven side effects of *M. oleifera* to this date [144].

## 6. Contraindications of *M. oleifera*

Despite the numerous positive possibilities associated with *M. oleifera* phytochemicals, there are suspicions that it contains harmful substances [22,145,146]. *M. oleifera* contains harmful chemicals such as alkaloids and other phytotoxins, which when consumed in high doses have potentially nerve-paralysing properties and other adverse effects [146]. Some of these phytochemicals include moringine, moringinine, estrogene, pectinesterase and phenols including tannin [22,145]. There are also unconfirmed reports that *M. oleifera* stems and roots potentially contain harmful phytochemical constituents, especially to pregnant women. Specifically, it is suspected that these elements of *M. oleifera* contain phytochemicals which have a potential of facilitating uterus contraction, leading to miscarriages in pregnant women. It is also suspected that it has the ability to prevent implantation in women, hence it has to be avoided by those attempting to conceive [147]. Some scientists suspect that the extracts from the roots have a potential of even causing paralysis and death. However, it is important to note that there are no major harmful effects of *M. oleifera* on humans that have been put forth by the scientific community to this date [144]. Based on the studies, and ongoing research, that has been conducted to date on both humans and animals, no adverse effects have been noted from *M. oleifera* products [144,148]. Although research is still ongoing, currently there are no scientifically confirmed toxic and harmful effects of *M. oleifera* extracts and products on both humans and animals. 

## 7. Water Purification

People living in developing and underdeveloped countries drink highly contaminated water [136]. It is estimated that at least 15% of the world’s population lacks safe/clean drinking water. It is estimated that water-related diseases kill more than 5 million people per annum globally [149]. The high cost of chemicals used to treat water has led most people in the rural communities of developing countries to rely on easily available and accessible water sources. Most of these sources are usually contaminated and are also suggested to contain waterborne diseases [150]. Thus the use of *M. oleifera* seeds, which are edible, as natural coagulants are now highly recommended because naturally occurring coagulants are suggested to be safe for human health [150,151].

*Moringa oleifera* seeds have been reported to contain coagulation properties [150,151], which are particularly recommended to use for high-turbid water (water with high levels of haziness or cloudiness) [136]. However, the coagulation activity on *M. oleifera* has been found to be low for low-turbid water [152,153]. When its seeds are dried, scarified, crushed and added to water, its powder acts as a coagulant which binds the microscopic colloidal particles and bacteria to form clump particles [154]. These particles settle at the bottom and the purified supernatant can be poured off [154]. Water treatment ranges from 2 seeds per 1 litre to 1 seed per 4 litres depending on the turbidity of the water [152]. *M. oleifera* seeds contain 1% active polyelectrolyte that neutralizes the negatively charged colloid mixture in the contaminated water [155]. There is a reduction in water conductivity, turbidity and total solids in treated water [156]. Ndhlala et al. [55] attribute the positive effects of *M. oleifera* through its antimicrobial properties against *Klebsiella pneumoniae, Staphylococcus aureus* bacteria and *Candida albicans* fungus. Furthermore, *M. oleifera* seed has been suggested to remove about 90–99% of the bacteria found in contaminated water [150].

While the use of *M. oleifera* in household water treatment is evident, its use of on a large scale is not detailed. Instead, in large scale water treatment plants, aluminium sulphate and potash are more commonly used as conventional chemical coagulants. Additionally, there have been suggestions of a secondary increase of bacteria after water coagulation, as well as the purified water containing some pathogenic germs or microorganisms [157]. In addition, the coagulant from *M. oleifera* is not available in pure or preserved form as it should be prepared fresh [157]. This limits its use and accessibility in areas where *M. oleifera* is not grown. *M. oleifera* increases the levels of organic matter in treated water, which may offset the colour, taste and odour of water, and these problems have the potential to worsen when treated water is stored for longer periods [153]. Due to the lack of adequate literature, we recommend further research on the toxicity in water purification used for human consumption, with possible implications for their large scale use.

## 8. Invasiveness and Allelopathy of *Moringa oleifera*

The coexistence of competitive invasive vegetation with native plants is crucial towards the long-term sustainable production of ecosystems [158]. Plants which are considered easily adaptable, as well as moderately invasive, have a high potential to impact the stability of ecosystems and their production of ecosystem services. For example, *M. oleifera* generally grows in most soil types, except for clayey soils, and grows well in harsh conditions in semi-arid and arid regions [159,160]. Consequently, there is a great concern that such a plant that easily grows in a wide range of conditions has the high potential to effortlessly become invasive [161]. There are conflicting reports about the extent of the invasiveness of *M. oleifera,* as it is regarded as potentially or moderately invasive, especially in the tropics [162], as it has growth attributes of forming dense thickets around the parent plant [163]. 

The Invasive Species Compendium (CABI) classifies *M. oleifera* as a low potential invasive species [164]. Comparable to other plant species with similar ecological growth attributes, it has been suggested to cause dire ecological consequences in the wet/dry tropics around the world [164]. For example, in northern Australia, it has escaped from gardens, leading to it being considered a minor weed in that region [163]. However, it may not necessarily be considered a problem plant in agricultural areas, as it colonises river banks because of the high water table all year round [163]. A study conducted in Trinidad and Tobago, which tested the invasiveness of *M. oleifera* using the Australian Weed Risk Assessment (WRA), concluded that the plant is a low-risk plant [155]. Instead, this study led to it being classified as a bioenergy crop in the Caribbean [161]. As a result of such conclusions, *M*. *oleifera* has been described as a naturalised plant crop in most of the countries in the tropics and subtropics [4].

Due to its growth characteristics and adaptability, there are contradicting results from the use of its extracts when investigating its potential to suppress or promote the growth of other vegetation. Some studies have reported positive effects of other plants grown after spraying leaf extracts from *M. oleifera*. For example, Fuglie [165], Mehboob et al. [166], Soliman et al. [167], Iqbal [168] and Nouman et al. [169] all reported positive effects of extracts from *M. oleifera* on plant growth characteristics of different crops. In Zambia, *M. oleifera* leaf extracts did not affect the time taken to germination of maize and wheat [170]. However, the leaf extract enhanced germination of sorghum resulted in delayed germination of wheat [170]. Some negative effects of extracts from *M. oleifera* plant parts have also been reported in parts of the world for both field and lab experiments. For instance, *M. oleifera* leaf extracts impeded the rate of germination of mungbean (*Vigna radiata* (L) Wilczek) under laboratory conditions, while root extracts also impeded its growth and yield under pot conditions in Bangladesh [171]. 

Elsewhere in Iraq, *M. oleifera* leaf, flower and seed extracts had negative effects on the seed germination, shoot and root growth of wild mustard (*Sinapis arvensis*) plants but were stimulatory to seed germination and growth of wheat (*Triticum aestivum* L.) seedlings [172]. In addition, *M. oleifera* leaves showed negative allelopathic effects on faba bean (*Vicia faba* L) growth in Saudi Arabia [167]. Several allelochemicals have also been found within different parts of the *M. oleifera* plant. For example, there are suggestions that the seeds and bark contain 4-(R-L-rhamnopyranosyloxy)-benzylglucosinolate while, 4-(R-L-rhamnopyranosyloxy)-benzylglucosinolate and benzyl glucosinolate have been isolated in *M. oleifera* roots [173]. However, the positive effects of the leaf extracts have been attributed to the rich naturally occurring cytokinin, along with phytohormones and inorganic salts that are in a naturally balanced concentration, as well as 4-(R-Lrhamnopyranosyloxy)-benzylglucosinolate, 3-caffeoylquinic acid *M. oleifera* leaves reportedly contain [173]. These combinations increase the yield of crops when applied exogenously [174].

The contradictory scientific publications regarding both the negative and positive effects of extracts from *M. oleifera* on co-existing vegetation make it difficult to certify it as a fully invasive plant. We came across no other records which classify *M. oleifera* as moderately or potentially invasive, except the already cited report in Australia [162]. Most studies on extracts from different *M. oleifera* plant parts (roots, leaves and stem) generally report positive effects on plant growth characteristics, and a low proportion reports negative effects. Therefore, introducing this species, especially in degraded ecosystems, should be done with care to avoid it colonizing native vegetation territory, since it easily adapts to any growth conditions.

## 9. Conclusions

This work sought to review the beneficial and adverse properties of *M. oleifera*. Specifically, the paper assessed the medicinal and nutritional properties and the ecological impact of the plant. Grounded in the findings of this work, after meticulously interrogating a plethora of studies, we conclude that:

- Gram on gram, *M. oleifera* contains higher amounts of elemental nutrients than most conventional vegetable sources which makes it a potentially lucrative crop to combat food and nutritional insecurity.
- There are no scientifically proven side effects of *M. oleifera* to this date, despite the lack of safety information on its utility, particularly in humans.
- Based on available *M. oleifera,* it produces a chemically diverse range of phytochemicals which can be exploited for the development of pharmaceutical agents.
- Due to a pool of phytochemicals found in *M. oleifera* extracts, a number of medicinal properties have been reported to date.
- *M. oleifera* holds great potential both as a food supplement and medicine, however, more clinical trials are needed for the development of pharmaceutical agents.
- *M. oleifera* has shown some potential as a water treatment agent and can be a useful resource particularly in resource poor communities.
- There is no literature that suggests that *M. oleifera* could be an invasive plant species, although extreme caution has to be exercised when replanting, or introducing it, particularly in degraded lands.

## Figures and Tables

**Table 1:** Properties of vitamins and minerals found in *Moringa oleifera* plant.

Bioactive Compound	Specific compound	Properties	References
Vitamin A	Retinol, Retinal and Retinoic acids	Leaves contain 11,300–23,000 IU (international unit) of vitamin A.	[5,59]
Vitamin B	Folates, such as 5-Formyl-5,6,7,8-tetrahydrofolic acid, 5,6,7,8-tetrahydrofolic acid and 5-Methyl-5,6,7,8-tetrahydrofolic acid	Involved in DNA synthesis and cell division.	[60]
Carotenoids	β-carotene	Ranges from 6.63 mg/100 g in fresh leaves to about 39.6 mg/100 g in air-dried leaves.	[4,61,62]
Vitamin C	Ascorbic acid	Found in amounts of about 200 mg/100 g (greater than in orange fruits). Acts as an antioxidant.	[63,64,65]
Minerals	Potassium (K), Iron (Fe), Calcium (Ca) and Magnesium (Mg).	Contains more calcium, iron and potassium than in milk, spinach and bananas respectively. Vegetative parts and immature fruits contain the most potassium.	[5,8,26]

**Table 2:** Some reported antioxidant properties of different plant parts of *M. oleifera.*

Antioxidant Model Used	Candidate Compounds	Solvent	Ref.
**Leaf**			
1,1-diphenyl 2-picrylhydrazyl (DPPH)	Crude extracts, quercetin, kaempferol, gallic, chlorogenic, ellagic, ferulic acid, rutin, gallic acid, vanillin	Water, 70% ethnol, 80% ethanol, 80% methanol. Chloroform, acetone	[80,82,84,85,86,87,89,90]
*β*-carotene-linoleic acid	Quercetin, kaempferol, gallic, chlorogenic, ellagic, ferulic acid, rutin, gallic acid, vanillin	Water, 70% ethnol, 80% methanol, chloroform	[80,82,85]
Superoxide radical scavenging	Quercetin, kaempferol	Water, 70% ethnol, 80% methanol	[80]
Liposome Peroxidation	Quercetin, kaempferol	Water, 70% ethnol, 80% methanol,	[80]
Enzymatic Lipid Peroxidation of Microsomes Induced by NADPH/ADP/Fe3+	Crude extracts, quercetin, kaempferol, gallic, chlorogenic, ellagic, ferulic acid, rutin	Water, 70% ethnol, 80% methanol, acetone, chloroform	[80,82,86]
Linoleic Acid Peroxidation System	Quercetin, kaempferol	Water, 70% ethnol, 80% methanol	[80]
Superoxide dismutase (SOD)	Crude extracts, quercetin, kaempferol, gallic, chlorogenic, ellagic, ferulic acid, rutin	Chloroform, water, 80% ethanol, acetone	[82,84,86,90]
Catalase	Crude extracts, quercetin, kaempferol, gallic, chlorogenic, ellagic, ferulic acid, rutin	Chloroform, water, 80% ethanol, acetone	[82,84,86,90]
Glutathione peroxidase	Crude extracts	Water, acetone	[86,90]
Nitric oxide (NO) radical scavenging	Crude extracts	Water, acetone	[86]
2,2′-azino-bis-3-ethylbenzothiazoline-6-sulphonic acid (ABTS)	Crude extracts	Water, acetone	[86]
Ferric Reducing Iron Power (FRAP)	Crude extract	Water, 80% ethanol, acetone	[85,86]
**Seed**			
UV accelerated method	Crude extract fractions	Chloroform/methanol (1:1), diethylether, n-butanol, and water	[83]
DPPH	Crude extract, gallic acid, chlorogenic acid, ellagic acid, ferulic acid, kaempferol, quercetin, vanillin.	Water, 80% ethanol	[84,85]
FRAP	Crude extract, gallic acid, chlorogenic acid, ellagic acid, ferulic acid, kaempferol, quercetin, vanillin	Water, 80% ethanol	[84,85]
SOD	Crude extract	80% ethanol	[84]
Catalase	Crude extract	80% ethanol	[84]
*β*-carotene-linoleic acid	gallic acid, chlorogenic acid, ellagic acid, ferulic acid, kaempferol, quercetin, vanillin	Water	[85]
Lipid Peroxidation	Crude extracts, gallic acid, chlorogenic acid, ellagic acid, ferulic acid, kaempferol, quercetin, vanillin	Water	[85,88,90]
**Flower**			
DPPH	Crude extract	80% ethanol	[84]
FRAP	Crude extract	80% ethanol	[84]
Superoxide dismutase (SOD)	Crude extract	80% ethanol	[84]
Catalase	Crude extract	80% ethanol	[84]
**Pod**			
DPPH	Crude extract, gallic acid, chlorogenic acid, ellagic acid, ferulic acid, kaempferol, quercetin, vanillin	Water, 80% ethanol	[84,85]
FRAP	Crude extract, gallic acid, chlorogenic acid, ellagic acid, ferulic acid, kaempferol, quercetin, vanillin	Water, 80% ethanol	[84,85]
SOD	Crude extract	80% ethanol	[84]
Catalase	Crude extract	80% ethanol	[84]
*β*-carotene-linoleic acid	Gallic acid, chlorogenic acid, ellagic acid, ferulic acid, kaempferol, quercetin, vanillin	Water	[85]
Lipid Peroxidation	Gallic acid, chlorogenic acid, ellagic acid, ferulic acid, kaempferol, quercetin, vanillin	Water	[85]
**Stem**			
DPPH	Crude extract	80% ethanol	[84]
FRAP	Crude extract	80% ethanol	[84]
SOD	Crude extract	80% ethanol	[84]
Catalase	Crude extract	80% ethanol	[84]
